# Combinatorial genetic analysis of a network of actin disassembly‐promoting factors

**DOI:** 10.1002/cm.21231

**Published:** 2015-08-22

**Authors:** Casey A. Ydenberg, Adam Johnston, Jaclyn Weinstein, Danielle Bellavance, Silvia Jansen, Bruce L. Goode

**Affiliations:** ^1^ Department of BiologyRosenstiel Basic Medical Science Research Center, Brandeis UniversityWalthamMassachusetts02454

**Keywords:** actin, actin disassembly, endocytosis, yeast, cell polarity, Cofilin, Coronin, Aip1, Twinfilin, GMF, Srv2/CAP1

## Abstract

The patterning of actin cytoskeleton structures in vivo is a product of spatially and temporally regulated polymer assembly balanced by polymer disassembly. While in recent years our understanding of actin assembly mechanisms has grown immensely, our knowledge of actin disassembly machinery and mechanisms has remained comparatively sparse. *Saccharomyces cerevisiae* is an ideal system to tackle this problem, both because of its amenabilities to genetic manipulation and live‐cell imaging and because only a single gene encodes each of the core disassembly factors: cofilin (*COF1*), Srv2/CAP (*SRV2*), Aip1 (*AIP1*), GMF (*GMF1/AIM7*), coronin (*CRN1*), and twinfilin (*TWF1*). Among these six factors, only the functions of cofilin are essential and have been well defined. Here, we investigated the functions of the nonessential actin disassembly factors by performing genetic and live‐cell imaging analyses on a combinatorial set of isogenic single, double, triple, and quadruple mutants in *S. cerevisiae*. Our results show that each disassembly factor makes an important contribution to cell viability, actin organization, and endocytosis. Further, our data reveal new relationships among these factors, providing insights into how they work together to orchestrate actin turnover. Finally, we observe specific combinations of mutations that are lethal, e.g., *srv2Δ aip1Δ* and *srv2Δ crn1Δ twf1Δ*, demonstrating that while cofilin is essential, it is not sufficient in vivo, and that combinations of the other disassembly factors perform vital functions. © 2015 The Authors. Cytoskeleton Published by Wiley Periodicals, Inc.

## Introduction

Cells have a finite pool of actin subunits from which they assemble a variety of filamentous arrays to perform different biological tasks. Many of these actin networks must undergo highly dynamic remodeling, which is achieved through coordinated actin assembly and disassembly mechanisms. In recent years, a relatively clear mechanistic picture has emerged for how filamentous actin arrays are assembled in cells, involving collaborations and interplay among various actin nucleation and elongation factors [Chesarone and Goode, 2009; Dominguez, 2009; Blanchoin and Michelot, 2012; Blanchoin et al., 2014]. Comparatively less is known about the cellular machinery and mechanisms driving the rapid disassembly of actin filament arrays. A set of six ubiquitous proteins (ADF/cofilin, Srv2/CAP, Aip1, GMF, coronin, and twinfilin) has emerged as a core set of actin disassembly machinery found in organisms as diverse as yeast and mammals (Ono, 2007; Poukkula et al., 2011; Brieher, 2013; Ono, 2013). However, among these six factors, only the functions and mechanisms of ADF/cofilin (referred to as cofilin hereafter) are well defined, and the roles of the other proteins and how they work in concert to disassemble actin networks are still not well understood.

Cofilin has been the focus of intense biochemical investigation for over 30 years [Harris and Weeds, 1983; Mabuchi, 1983; Nishida et al., 1984; Nishida et al., 1985; Cooper et al., 1986; Yonezawa et al., 1988; Andrianantoandro and Pollard, 2006; Suarez et al., 2011]. Cofilin binds cooperatively to the sides of filaments and severs them, creating new ends to accelerate either assembly or disassembly, depending on conditions (e.g., monomer concentration and efficiency of barbed end capping) [Cooper et al., 1986; Bravo‐Cordero et al., 2013]. More recently, Srv2/CAP, Aip1, coronin, GMF, and twinfilin have each been implicated in the actin disassembly process via genetic and/or biochemical observations. Srv2/CAP has two separate functions, one in enhancing cofilin‐mediated severing of filaments [Normoyle and Brieher, 2012; Chaudhry et al., 2013; Zhang et al., 2013] and one in recycling cofilin and ADP‐actin monomers [Moriyama and Yahara, 2002; Balcer et al., 2003; Mattila et al., 2004; Chaudhry et al., 2014; Jansen et al., 2014]. Aip1 promotes cofilin‐mediated actin filament disassembly both by enhancing severing and by capping newly generated barbed ends [Okada et al., 1999; Rodal et al., 1999; Okada et al., 2002; Balcer et al., 2003; Ono et al., 2004; Brieher et al., 2006; Okada et al., 2006; Kueh et al., 2008; Nadkarni and Brieher, 2014; Gressin et al., 2015; Jansen et al., 2015]. Coronin works together with cofilin and Aip1 to promote disassembly even under assembly conditions, by accelerating cofilin recruitment to filament sides and enhancing severing and capping [Brieher et al., 2006; Cai et al., 2007a; Gandhi et al., 2009]. GMF is an ADF‐homology (ADFH) domain protein that binds Arp2/3 complex and catalyzes filament debranching [Gandhi et al., 2010b; Luan and Nolen, 2013; Ydenberg et al., 2013; Poukkula et al., 2014; Haynes et al., 2015]. Twinfilin consists of ADFH domains connected by a short linker, and although it has been primarily described as an actin monomer sequestering protein [Goode et al., 1998; Ojala et al., 2002], genetic interactions with cofilin in yeast and flies implicate twinfilin in promoting actin disassembly [Goode et al., 1998; Wahlstrom et al., 2001].

A major challenge now is to gain a deeper understanding of the individual and combined functions of these six disassembly factors, by investigating their physical interactions, biochemical activities, loss‐of‐function phenotypes, and genetic relationships. However, these goals have been hampered by the complexity of analyzing whole sets of proteins or genes. Here, we focus on improving our understanding of the genetic interactions and shared in vivo functions of the less‐studied core actin disassembly factors in *Saccharomyces cerevisiae*. Budding yeast provides an attractive model system for this analysis because of the low complexity of its genome, e.g., mammals have three cofilin genes, seven coronin genes, two twinfilin genes, two Srv2/CAP genes, and two GMF genes, whereas *S. cerevisiae* has only a single gene encoding each of these factors [Morgan and Fernandez, 2008; Poukkula et al., 2011]. In addition, yeast readily enables the simultaneous disruption of multiple genes and the analysis of their effects on the actin cytoskeleton.

Yeast cells contain three prominent F‐actin structures: cortical patches, which are sites of endocytosis; cables, which are tracks for myosin‐based transport of vesicles and organelles required for polarized cell growth; and cytokinetic rings, which facilitate cell division [Adams and Pringle, 1984; Moseley and Goode, 2006; Mishra et al., 2014]. Each of these F‐actin structures is highly dynamic, with patches and cables turning over in 10–20 s. Regulated disassembly plays a vital role in vivo, as demonstrated by the lethality caused by deleting the yeast cofilin gene (*COF1*) [Moon et al., 1993]. Somewhat perplexingly though, deletions of other genes in the disassembly ensemble have little if any effect on yeast cell growth on their own, e.g., *crn1Δ*, *aip1Δ*, *twf1*Δ, and *gmf1*Δ, yet these mutations each can strongly exacerbate the temperature sensitivity of *cof1* partial loss‐of‐function alleles, and in some cases cause lethality [Goode et al., 1998; Goode et al., 1999; Rodal et al., 1999; Gandhi et al., 2010b]. Together, these observations suggest that more information is needed to understand their roles and contributions. Importantly, these mutations have never been analyzed side‐by‐side with an isogenic strain set, or in combination sets beyond simple pairs. Here, we performed a systematic analysis for the first time of single, double, triple, and quadruple mutant strains, analyzing them by fixed and live‐cell imaging for defects in cell growth, actin organization, and endocytosis.

## Results

### Shared Essential Functions Among Actin Disassembly Genes

**Figure 1:**
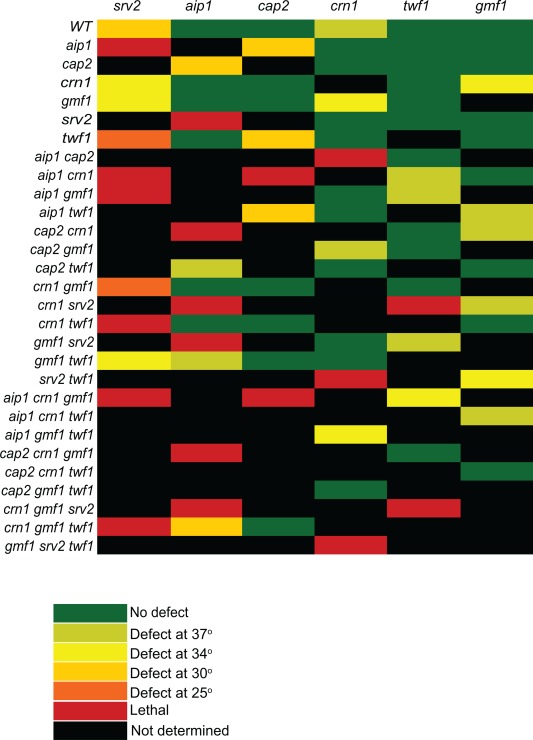
Growth defects due to loss of actin disassembly genes. Growth curves of yeast strains were performed in quadruplicate in 96‐well plates grown in YPD medium. The data show the effect that each deletion (indicated along the top axis) has when added to the mutation(s) indicated along the left axis. Raw growth rates and strain numbers are shown in Supporting Information, Table S2. In the heat map, a defect indicated at a given temperature was considered to have occurred if the doubling time was increased by at least twofold. Synthetic lethality, indicated in red, was determined from tetrad analysis.

We first generated a large set of single, double, triple, and quadruple mutant strains combining null mutations in *AIP1*, *CRN1*, *GMF1*, *SRV2*, and *TWF1* (details of strain construction in Supporting Information, Table S1). Because some of these proteins have been proposed to work at least in part by capping severed ends of filaments, we also included a null mutation in *CAP2*, which encodes one of the two subunits of yeast capping protein (note that *cap2Δ* abolishes capping protein function [Kim et al., 2004]). We also note that it was not possible to create strains with certain combinations of mutations due to synthetic lethality (Fig. 1, red boxes). This analysis confirmed previously reported synthetic lethal interactions between *srv2Δ* and *aip1Δ* and synthetic slow‐growth interactions between *aip1Δ* and *cap2Δ* [Balcer et al., 2003; Michelot et al., 2013]. Importantly, the further deletion of *CRN1* in the *aip1Δ cap2Δ* background resulted in lethality, consistent with the view that Aip1 and coronin function together in capping filaments after severing [Brieher et al., 2006; Kueh et al., 2008; Ishikawa‐Ankerhold et al., 2010; Jansen et al., 2015]. The *crn1Δ srv2Δ twf1Δ* triple mutant was also lethal, revealing another important essential function shared among a specific set of disassembly factors.

For all the viable mutant strains, we measured doubling times during logarithmic growth phase in rich medium at 25, 30, 34, and 37°C. We used these data to determine the specific effect of deleting each gene in a strain already containing zero, one, two, or three deletions in other disassembly factors (Fig. 1 and Supporting Information, Table S2). We expressed the results as the minimum temperature at which at least a twofold change in the doubling time was observed relative to the parent strain. Among the single mutants, *srv2Δ* had a strong effect on growth at multiple temperatures, *cap2Δ* affected growth only at elevated temperatures, and *aip1Δ*, *gmf1Δ*, and *twf1Δ* each grew as well as wild type at all temperatures. On the other hand, *crn1Δ* single mutants caused a slight growth defect at 37°C, which was not previously observed [Heil‐Chapdelaine et al., 1998; Goode et al., 1999; Gandhi et al., 2010a], suggesting that our quantitative growth assays may be more sensitive than comparing growth on plates.

In the context of strains already lacking one or more disassembly components, stronger defects were apparent after deleting additional genes. In particular, lethality or strong growth defects were observed when *srv2Δ* was combined with other disassembly mutants. For analysis of mutations not involving *srv2Δ*, a number of specific combinations led to clear growth defects, including *aip1Δ cap2Δ* and *gmf1Δ crn1Δ*. This suggests that the process of actin disassembly involves multiple factors with unique, yet partially redundant functions. It also demonstrates that the severing activity of Cof1 is not the only activity among this group of proteins required for viability. Instead, it is apparent that there are specific combinations of activities among the remaining components that are essential and additional combinations that are required for normal growth.

### Contributions of *Aip1*, *Crn1*, *Gmf1*, and *Twf1* in Regulating Endocytic Patch Dynamics

In yeast, branched actin filament arrays are assembled at sites of endocytosis and play an essential role in driving invagination and scission of vesicles at the cell cortex [Kaksonen et al., 2003; Idrissi et al., 2012; Kukulski et al., 2012]. Live‐cell imaging studies have defined a system of >60 proteins recruited to patches in a defined temporal order to orchestrate this process [Kaksonen et al., 2006; Galletta et al., 2010; Boettner et al., 2012]. Actin and actin‐associated proteins are among the last proteins to arrive, and in wild‐type cells, the duration of this “late actin phase” is only 15–20 s before vesicles are internalized [Kaksonen et al., 2003; Kaksonen et al., 2005]. Previous studies have shown that *crn1Δ, aip1Δ* and *cof1* alleles each individually extend the lifetime of the actin phase [Okreglak and Drubin, 2007; Lin et al., 2010; Okreglak and Drubin, 2010; Liu et al., 2011], although the effects of *aip1Δ* and *crn1Δ* were modest, whereas *srv2Δ* and *twf1Δ* do not strongly affect patch dynamics [Kaksonen et al., 2005], and *gmf1Δ* effects are unexplored.

**Figure 2:**
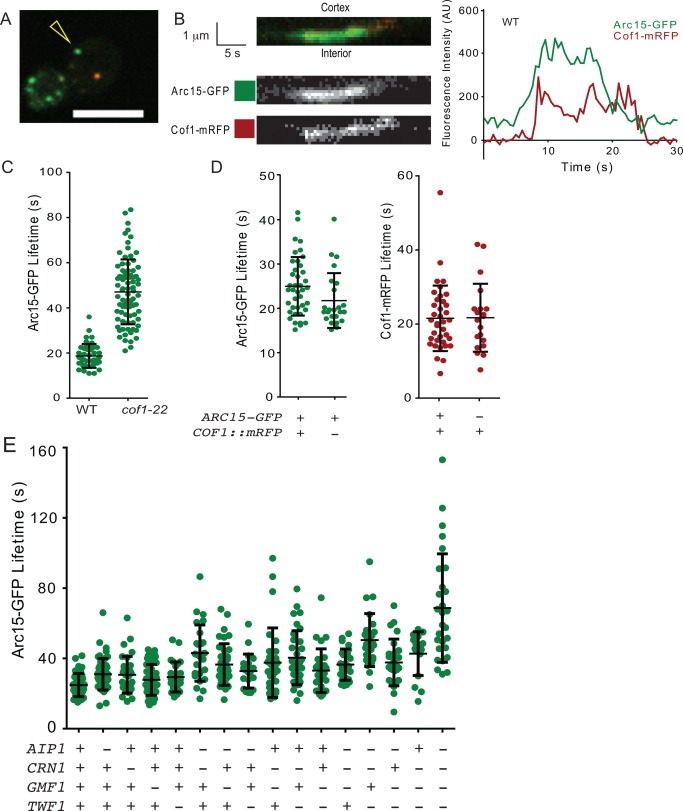
Actin patch maturation is delayed in disassembly mutants. (A) Still image from Supporting Information, Movie S1. Arc15‐GFP and Cof1‐mRFP were imaged at 0.5 s intervals. Scale bar = 5 μm. (B) Example kymograph and intensity profile of the wild type actin patch, designated in (A) by yellow arrowhead. Left, *top row*: merged channels. Scale bar = 1 μm (*y*‐axis), 5 s (*x*‐axis). *Left, middle row*: Green (Arc15‐GFP) channel. *Left, bottom row*: Red (Cof1‐mRFP) channel. *Right*: Intensity profile of the kymograph. Background was subtracted as described in the section titled Materials and Methods. (C) Effect of *cof1‐22* mutation on Arc15‐GFP lifetime. Strains: left, DDY2752 (wild type); right, CY384 (*cof1‐22*). Unlike the other experiments in this figure, these strains do not express *COF1::mRFP*. (D) Comparison of average Arc15‐GFP lifetime in strains with and without the *COF1::mRFP* plasmid. Strains: left, CY259 (data is the same as in Fig. 2E); right, same mother strain (DDY2752) transformed with empty vector, pRS415 [Sikorski and Hieter, 1989]. The difference in average Arc15‐GFP lifetime between the two strains was not significant (*p* = 0.06, Student's *t*‐test). Comparison of average Cof1‐mRFP lifetime in strains with and without the Arc15‐GFP marker. Strains: left, CY259; right, equivalent strain without Arc15‐GFP (DDY904 transformed with pBJ1807 *COF1::mRFP*). The difference between the two strains is not significant (*p* = 0.95, Student's *t*‐test). (E) Arc15‐GFP lifetime in the indicated mutants (from left to right: yeast strains CY259, CY262, CY303, CY260, CY282, CY307, CY261, CY280, CY304, CY305, CY279, CY306, CY310, CY281, CY309, CY308; *n* ≥ 21). Points in the graph represent lifetimes of individual patches, and black brackets represent the population mean and standard deviation. Results are also listed in Supporting Information, Table S3.

To monitor the kinetics of the actin phase, we used a dual‐color imaging system in strains carrying different combinations of deletions in *AIP1*, *CRN1*, *TWF1*, and *GMF1* (Fig. 2A and B, and Supporting Information, Movie S1). Because GFP tags on actin itself interfere with function, we used Arc15‐GFP (ARPC5), the smallest subunit of the Arp2/3 complex as a proxy, given that it is an integral component of branched F‐actin networks. This tagged protein is fully functional, and shows the same kinetic behavior as the commonly used Abp1‐GFP marker [Kaksonen et al., 2003]. As reported previously for Abp1‐GFP [Okreglak and Drubin, 2007; Lin et al., 2010], we found that the hypomorphic *cof1‐22* allele extended the patch lifetime of Arc15‐GFP (Fig. 2C), showing that compromised actin turnover extends the patch lifetime. We also included Cof1‐mRFP in our analysis because we were interested in the kinetics of disassembly. In wild type cells, this marker arrives at patches shortly after the first appearance of actin and Arp2/3 complex, and persists there while vesicles are internalized [Okreglak and Drubin, 2007; Lin et al., 2010]. Cof1‐mRFP is an internal in‐frame tag and complements much though not all of *COF1* function, as demonstrated by Lin et al. [2010]; it is reported to be the most functional tag available for Cof1, and we used it only in strains that also contain endogenous *COF1*. In wild type cells, we observed Arc15‐GFP and Cof1‐mRFP lifetimes similar to those previously reported, and the behavior of each tag was not significantly affected by the presence of the other (Fig. 2D).

Cof1‐mRFP arrived at patches shortly after actin (marked by Arc15‐GFP), and persisted there for the lifetime of the patches at the cortex (Fig. 2B). Both markers were internalized and could sometimes be observed inside the cell in the same focal plane briefly before disappearing. The average lifetime of Arc15‐GFP in wild‐type cells was 24.8 ± 6.6 s (mean ± SD; Fig. 2D; Supporting Information, Table S3), and did not change substantially in any of the single mutants (*aip1Δ* = 31.0 ± 9.0 s; *crn1*Δ = 30.7 ± 10.5 s; *gmf1Δ* = 27.8 ± 8.8 s; *twf1*Δ =29.4 ± 8.6 s). However, the lifetimes became successively longer in double and triple mutants, and were longest in the quadruple *aip1Δ crn1Δ gmf1Δ twf1Δ* mutant, with a mean lifetime 68.7 ± 30.9 s. Notably, this is even longer than the actin lifetime in *cof1‐22* mutants (Fig. 2C).

To identify activities that may be impacted by the progressive loss of specific sets of disassembly factors, for each pair of genes, we compared the actin phase lifetimes of the individual single mutants with the double mutants. If each gene makes a separate contribution to extending patch lifetime, then the extension of lifetime in the double mutant (compared to wild type) should be close to the sum of the lifetime extensions of the two single mutants. To accomplish this, we performed a z‐test, comparing this hypothetical, additive, double mutant to the real double mutant lifetime population (Table 1). Two double mutants (*aip1Δ crn1Δ* and *crn1Δ twf1Δ*) were more severely impaired than expected for the sum of their individual defects, suggesting that these pairs of proteins may have more closely related functions in disassembly.

**Table 1:** Actin Patch Lifetime in Double Mutants

Genotype	Observed lifetime (s)	Expected lifetime (s)	*p*
Wild‐type (WT)	24.8		
*aip1*Δ	31.0		
*crn1*Δ	30.7		
*gmf1*Δ	27.5		
*twf1*Δ	29.4		
*aip1*Δ *crn1*Δ	44.0	36.8	0.025*
*aip1*Δ *gmf1*Δ	36.5	33.6	0.066
*aip1*Δ *twf1*Δ	32.8	35.6	0.904
*crn1*Δ *gmf1*Δ	37.6	33.3	0.123
*crn1*Δ *twf1*Δ	40.4	35.3	0.028*
*gmf1*Δ *twf1*Δ	33.0	32.1	0.341

*p < 0.05.Observed lifetimes are averages (*n* > 21; data in Supporting Information, Table S3). Expected lifetime was calculated using the equation: (mutant1 − WT) + (mutant2 − WT) + WT = mutant1 + mutant2 − WT, which assumes that each individual mutation independently adds a discrete amount of patch lifetime to the base WT lifetime. For example, *aip1*Δ adds 31.0 − 24.8 = 6.2 s to WT, *crn1*Δ adds 30.7 −24.8 = 5.9 s, and the double mutant would be expected to add the sum of these to the WT lifetime (24.8 + 6.2 + 5.9 = 36.9 s). The *p*‐value was determined by a z‐test between the double mutant population and the expected value.

### Loss of Disassembly Factors Alters *Cof1* Dynamics at Endocytic Patches

**Figure 3:**
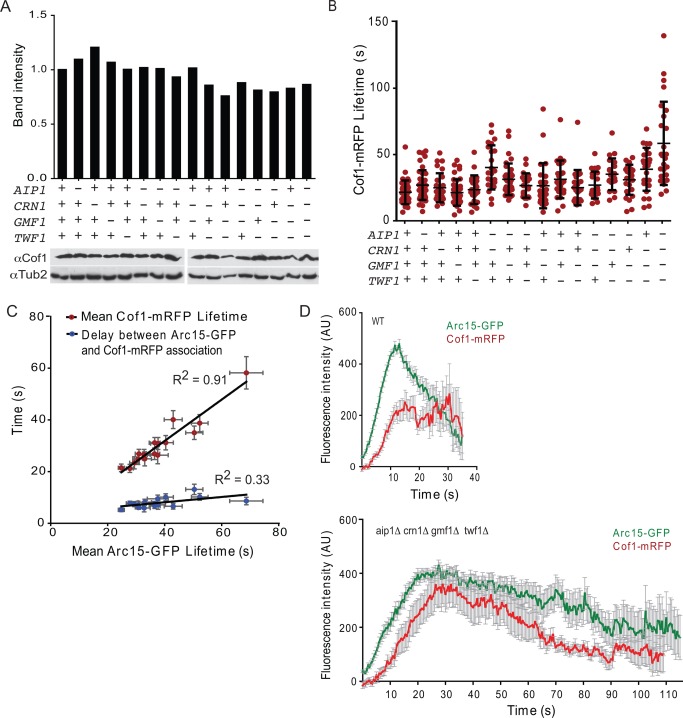
Cofilin persists longer on actin patches in disassembly mutants. (A) Cof1 levels in the indicated strains were quantified by western blot analysis with anti‐Cof1 antibodies. Strains (left to right): CY4, CY54, CY328, CY38, AJY13, CY332, CY56, AJY35, CY326, CY315, AJY39, CY334, CY317, AJY42, CY319, CY321. Data shown (top) are the average from two experiments. For western blots (example shown in lower panel), the intensity of the Cof1 signal was divided by the intensity of the loading control (Tub2) band, and then normalized to the wild type strain. (B) Cof1‐mRFP lifetimes in the same 16 yeast strains as in Fig. 2E. Each point in the graph represents the Cof1‐mRFP lifetime of an individual patch, and black brackets represent mean and standard deviation. Results are also listed in Supporting Information, Table S3. (C) Correlation between average Arc15‐GFP lifetime and average Cof1‐mRFP lifetime for each strain, from data in Figs. 2E and 3B. Red circles are the correlation between Arc15‐GFP lifetime and Cof1‐mRFP lifetime, and blue circles are the delay between the first appearance of Arc15‐GFP and the first appearance of Cof1‐mRFP. One data point is plotted for each mutant, but was calculated from a set of observations (see the section titled Materials and Methods), and error bars represent the SEM. A trend line was fit using the least‐squares method. (D) Arc15‐GFP and Cof1‐mRFP profiles in WT (CY259) and *aip1Δ crn1Δ gmf1Δ twf1Δ* (CY308). Each profile is an average of 52 observations for WT and 27 observations for the quadruple mutant. Error bars represent SEM.

We next considered how deletions in the core disassembly factors affected the expression levels of endogenous Cof1 and/or the dynamics at actin patches of Cof1‐mRFP (expressed from a plasmid in these strains). Quantitative western blotting showed that all of our strains had wild‐type levels of endogenous Cof1 (Fig. 3A). Cof1‐mRFP lifetimes increased progressively after deletion of the other disassembly components, ranging from 21.5 ± 8.9 s in wild type cells to 58.2 ± 31.3 s in *aip1Δ crn1Δ gmf1Δ twf1Δ* cells (Fig. 3B). This trend paralleled the increase in actin/Arc15‐GFP lifetimes in the same mutant strains, and indeed, there was a tight correlation between mean Arc15‐GFP lifetimes and Cof1‐mRFP lifetimes in the 16 strains (Fig. 3C; *R* ^2^ = 0.91).

We also examined how these mutations affected the length of the earliest part of the actin phase when the actin network is being rapidly formed by Arp2/3 complex but the disassembly factors have not yet arrived [Okreglak and Drubin, 2007; Lin et al., 2010]. The delay between arrival of Arc15‐GFP and Cof1‐mRFP increased from 5.2 ± 4.2 s in wild type cells to 8.6 ± 7.3 s in *aip1Δ crn1Δ gmf1Δ twf1Δ* cells, and again correlated with extended actin lifetimes (Fig. 3C; *R* ^2^ = 0.33). These data show that loss of some disassembly factors can extend the “actin assembly phase,” possibly due to reduced replenishment of the actin monomer pool, and/or delay the arrival of other disassembly factors such as cofilin.

We also examined Cof1‐mRFP kinetics at patches by comparing the timeline profiles for average intensity of Arc15‐GFP and Cof1‐mRFP between wild‐type and mutant cells. This was achieved by aligning all of the profiles for a given strain, starting from the first appearance of Arc15‐GFP, and calculating the average intensity of Arc15‐GFP and Cof1‐mRFP at each time point (Fig. 3D). Two major differences between wild type and mutants became evident. First, Arc15‐GFP signal accumulated more rapidly and peaked by 10 s in wild type cells, but not until almost 20 s in *aip1Δ crn1Δ gmf1Δ twf1Δ* cells. Second, in wild‐type cells, Cof1‐mRFP peaked by about 12 s and remained high even as Arc15‐GFP eventually declined, whereas in mutant cells Arc15‐GFP and Cof1‐mRFP signals declined in parallel. Put another way, in wild‐type cells, the ratio of Arc15‐GFP to Cof1‐mRFP changed drastically during patch maturation, as previously reported (Okreglak and Drubin, 2007; Lin et al., 2010), suggesting that as patches mature, more and more Cof1 accumulates relative to the amount of F‐actin remaining. In contrast, in the severe disassembly mutants, the ratio of Cof1‐mRFP to Arc15‐GFP remained more constant. Thus, one important function of the other disassembly proteins may be to facilitate the steady enrichment of Cof1 on actin patches.

### Actin Organization Defects and Aberrant *Cof1* Decoration of Actin Cables in Disassembly Mutants

**Figure 4:**
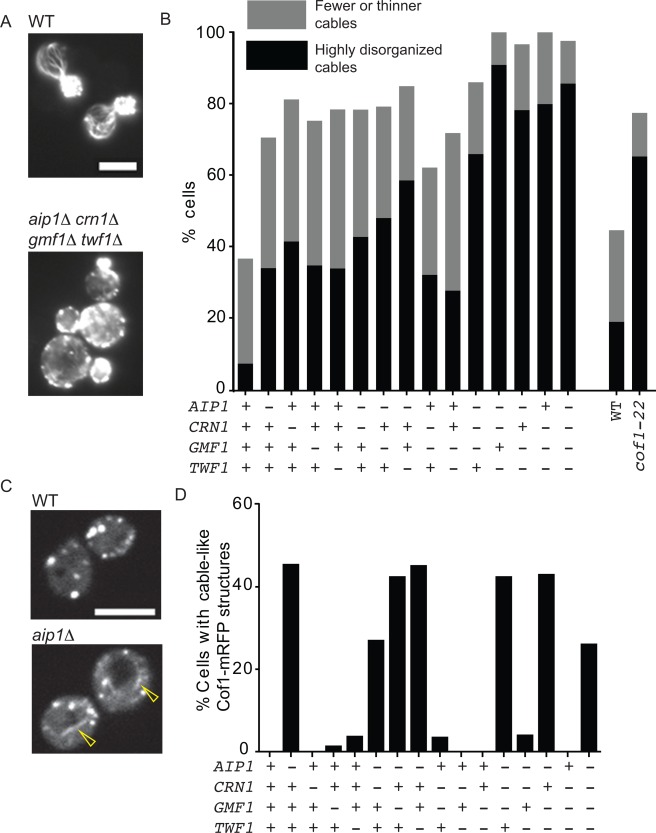
Disassembly mutants lead to defects in actin cable architecture and cell polarity. (A) Sample images of cells fixed and stained with rhodamine–phalloidin. Scale bar, 5 μm. (B) Polarity analysis in the indicated strains; categories as in [Graziano et al., 2011]. Scoring of cells (*n* ≥ 33) was performed in a double‐blind manner as described in the section titled Materials and Methods. Strains (left to right): DDY904, CY54, CY45, CY38, AJY13, CY68, CY56, AJY36, CY41, AJY39, CY70, AJY41, AJY42, AJY44, AJY46. In a separate experiment, WT (DDY904) and *cof1‐22* (PLY29) were compared (*n* ≥ 98 cells each). (C, D) Appearance of cofilin‐decorated cable‐like structures in disassembly mutants. Example images of Cof1‐mRFP in WT (CY259) and *aip1* (CY262) strains (C), with yellow arrowhead pointing to a cable‐like, Cof1‐mRFP‐decorated structure. The percentage of cells with such structures was scored in the indicated strains (D), and was found to correlate closely with the loss of *AIP1*. Strains are the same as in Fig. 2.

Disassembly mutants such as *srv2Δ* and *cof1‐22* have been shown to cause strong morphological and polarization defects, including enlarged cell size, depolarized actin patches, and diminished actin cable staining [Lappalainen and Drubin, 1997; Balcer et al., 2003]. To test whether such defects were also present in the mutant strains generated here, we fixed and stained cells with rhodamine–phalloidin (Fig. 4A). Quadruple mutants had severely depolarized actin cytoskeletons, exhibiting many of the same defects described for *srv2*Δ and *cof1‐22* cells, including large cell size, “brighter” and depolarized actin patches, and strongly reduced cable staining (Fig. 4B). Partial defects were apparent in each of the four single mutants, and were progressively more severe in combinatorial mutants. Thus, individual single mutants each cause minor defects in actin organization that may have been overlooked in previous studies. Furthermore, the extent of depolarization in the quadruple mutant was greater than in *cof1‐22* (Fig. 4B).

Cof1 has been observed to accumulate on actin cables in *aip1*Δ cells, but not on cables in wild‐type cells [Rodal et al., 1999; Okada et al., 2006; Lin et al., 2010]. Consistent with these observations, we observed Cof1‐mRFP cable‐like staining in all double, triple, and quadruple strains that included *aip1Δ*, but no mutants that were *AIP1^+^* (Fig. 4C and D, and Supporting Information, Movie S2). Further, time lapse imaging revealed that Cof1‐mRFP‐decorated cables in *aip1Δ* cells persisted for an average of 58.8 ± 40.8 s. While it is not possible to directly compare lifetimes of Cof1‐mRFP decoration in wild‐type cells, estimates for cable turnover rates based on extension rates and lengths are 5–10 s [Yu et al., 2011]. In *aip1Δ crn1Δ gmf1Δ twf1Δ* cells, lifetimes of Cof1‐mRFP‐decorated cables were similar to *aip1Δ* single mutants (57.3 ± 21.9 s), reinforcing the view that these defects arise from the loss of *AIP1*. Together, these observations demonstrate that *aip1Δ* leads to formation of stabilized cables abnormally decorated with Cof1, which is consistent with recent in vitro observations showing that Aip1 prevents cofilin from overdecorating and hyperstabilizing filaments [Nadkarni and Brieher, 2014; Gressin et al., 2015; Jansen et al., 2015].

## Discussion

Many of the most important functions performed by cellular actin structures depend on the filaments comprising these networks being assembled and disassembled on very short timescales. How such rapid actin filament turnover dynamics are achieved is only beginning to be understood, but appears to involve the filament severing protein cofilin working in concert with a core set of actin disassembly cofactors that includes Srv2/CAP, Aip1, coronin, GMF, and twinfilin. While recent studies have begun to define how each of these proteins functions biochemically, our understanding of how they work together in vivo to drive rapid actin turnover has been hampered in part by limited genetic analysis on the network. Studies in a variety of systems have begun to tackle this problem by analyzing the effects of disrupting pairs of disassembly factors [Ishikawa‐Ankerhold et al., 2010; Lin et al., 2010; Berro and Pollard, 2014; Poukkula et al., 2014; Talman et al., 2014]. Here, we leveraged the genetic amenability of *S. cerevisiae* to more extensively and combinatorially analyze single, double, triple, and quadruple mutations of disassembly factors for their effects on cell growth, actin cytoskeleton organization, and dynamics of endocytosis. Overall, our results demonstrate that each disassembly factor makes a unique contribution to these processes in vivo. Further, our data provide new insights into genetic relationships among disassembly factors and define specific combinations that are essential for viability, as discussed below.

Genetic interactions between *aip1Δ* and *cap2Δ* have been reported [Balcer et al., 2003; Michelot et al., 2013; Berro and Pollard, 2014], and we observed similar growth defects for *aip1Δ cap2Δ* mutants. These observations agree well with biochemical studies showing that Aip1 caps the barbed ends of filaments severed by cofilin [Okada et al., 2002; Balcer et al., 2003; Jansen et al., 2015]. Further, we observed that *aip1Δ crn1Δ* double mutants have a synthetic defect in Arc15‐GFP patch lifetime (Table 1), and that *aip1Δ cap2Δ crn1Δ* triple mutants are lethal. Together, these results point to a role for coronin in capping filament ends in vivo, which agrees with recent single molecule analyses in vitro [Jansen et al., 2015]. This is also consistent with studies showing that cofilin, Aip1, and coronin work together biochemically to rapidly disassemble actin filaments even under assembly‐promoting conditions [Brieher et al., 2006; Kueh et al., 2008], and with genetic studies from other organisms showing that simultaneously disrupting Aip1 and coronin leads to synthetic defects in actin‐based processes [Ishikawa‐Ankerhold et al., 2010; Lin et al., 2010; Talman et al., 2014].

We also observed that *crn1Δ twf1Δ srv2Δ* triple mutants are lethal, and that *crn1Δ twf1Δ* double mutants had synthetic defects in cell growth and Arc15‐GFP patch lifetime. Since the mechanistic role of twinfilin in actin disassembly is unclear, why this particular combination of three mutants (*crn1Δ*, *twf1Δ*, and *srv2Δ*) is lethal cannot yet be explained, but should inspire future biochemical analysis of the individual and combined effects of these proteins on actin disassembly.

Gmf1 showed the weakest phenotype of the disassembly factors. It tended to cause clear defects only when multiple other components were already missing. However, one exception to this trend was the *crn1Δ gmf1Δ* double mutant, which grew worse than the already compromised *crn1Δ* strain. GMF and coronin each promote actin filament debranching, inhibit actin nucleation by Arp2/3 complex [Humphries et al., 2002; Cai et al., 2008; Nakano et al., 2010; Gandhi et al., 2010b; Haynes et al., 2015], and stabilize the open (inactive) conformation of Arp2/3 complex [Rodal et al., 2005; Ydenberg et al., 2013]. Thus, GMF and coronin may have coordinated effects on Arp2/3 complex, which together are required for robust turnover of branched actin filament networks.

Our data show that the most sensitive readout of impaired actin disassembly in vivo is cell polarization, and indeed the only clear defect observed in all of our single mutants was a partial depolarization of actin cables and patches. Polarized cable formation depends on the ongoing recruitment of formins at the bud cortex, and the polarity factors that recruit and activate formins at the bud tip are themselves dynamically maintained at this position through targeted delivery on cables to counteract dispersion by actin‐dependent endocytosis [Irazoqui et al., 2005; Burston et al., 2009]. Given this sensitive requirement for two of the major actin systems (patches and cables), perhaps it not surprising that small alterations in actin turnover dynamics can disrupt polarity. On the other hand, the same single mutations cause only very subtle defects in endocytosis, as measured by patch lifetime analysis. We consider three possibilities for this apparent paradox. First, actin coats remain associated with endocytic vesicles after scission and inward movement, and coat disassembly may be a crucial step in downstream events of the endocytic pathway, as previously hypothesized in studies on *cof1* hypomorphic alleles [Okreglak and Drubin, 2007]. Second, small differences in endocytic efficiency may add up, leading to larger defects in polarity. Third, the rate of disassembly of actin cables may be more sensitive than that of patches to deletions in some of the disassembly factors. Indeed, *cof1‐22*, *crn1Δ*, and *aip1Δ* single mutants each cause more severe defects in actin cable turnover than actin patch turnover [Okada et al., 2006; Gandhi et al., 2009].

Finally, we note that many of our mutants prolonged not only the later stages of endocytic patch kinetics when disassembly factors are present on the patch, but also the earlier assembly phase (Fig. 3). One likely explanation for the prolonged assembly phase is that loss of disassembly factors decreases the rate of actin monomer recycling, thus slowing new assembly. Indeed, *cof1* hypomorphic alleles reduce the rate of in vivo actin filament treadmilling, presumably because impaired disassembly reduces the monomer pool and impedes new polymerization [Okreglak and Drubin, 2007]. Another possibility is that the delay between onset of actin assembly and arrival of cofilin results from an absence of disassembly cofactors that normally help recruit cofilin to patches, e.g., coronin [Jansen et al., 2015].

In summary, our results demonstrate that each disassembly factor makes a unique genetic contribution to actin turnover in vivo, and given their conservation suggests that this network of proteins has been optimized and maintained across evolution, from yeast to humans. The importance of these same proteins in multicellular organisms is demonstrated by the requirement for Aip1, CAP1, coronin, GMF, and twinfilin in maintaining proper lamellipodial dynamics [Rogers et al., 2003; Bertling et al., 2004; Cai et al., 2007b; Iwasa and Mullins, 2007; Aerbajinai et al., 2011; Poukkula et al., 2014; Haynes et al., 2015], and the requirement for many of these same disassembly factors in intercell spreading of *Listeria* [Talman et al., 2014]. In addition, our data show unequivocally that cofilin is not sufficient to support rates of disassembly required for viability, because specific combinations of the other disassembly factors are lethal. Overall, this paints a new picture of the regulation of actin disassembly, in which at least six conserved proteins work in concert as components of an integrated network promoting rapid and efficient filament disassembly.

## Materials and Methods

### Yeast Media, Strains, and Plasmids

Yeast strains along with a brief description of their construction methods are provided in Supporting Information, Table S1. Yeast media and transformation procedures were performed as described in Adams et al. [1997]. The *srv2Δ::HIS3, cap2Δ::HIS3, crn1Δ::NatMX6* and *twf1Δ::HIS3* knockout constructs were created by the Longtine method [Longtine et al., 1998]. The *aip1Δ::URA3* knockout construct was made by amplifying the *AIP1* locus containing the disruption from DBY6527 and introducing this mutation into our wild‐type strain [Amberg et al., 1995]. The *crn1::LEU2* knockout construct was made similarly by amplifying the *CRN1* locus containing the disruption from DDY1518 [Goode et al., 1999]. The *gmf1Δ::kanMX6* knockout construct was made by amplifying the *GMF1/AIM7* locus containing the disruption from BGY3090 [Gandhi et al., 2010b]. Knockouts were confirmed by PCR across the affected region, and comparing the sizes of the resulting products with controls. In the case of *aip1*Δ::*URA3*, the PCR product was digested with NcoI to distinguish wild‐type and mutant products. Swapping of *crn1::LEU2* with *crn1Δ::NatMX6* was confirmed by ensuring the resulting strain was Leu2^−^.

### Growth Analysis

Growth curves were performed in a multi‐well plate reader (Tecan, San Jose, CA) as described in Ydenberg et al. [2013]. Doubling time was measured in the logarithmic phase of growth, and was averaged between replicate wells. Experiments were performed separately at four different temperatures (25, 30, 34, and 37°C). A table of growth rate ratios and a heat map of the temperature needed to see a twofold increase in doubling time were prepared using a Python script, provided at https://github.com/CAYdenberg/Actin-disassembly–yeast‐growth‐rate‐analysis.

### Live‐Cell Imaging

All images were acquired using an upright microscope (Ni‐E; Nikon) equipped with a spinning disc head (CSU‐W1; Yokugawa Corporation of America), a 100× NA 1.45 Plan Apochromat objective, and an electron multiplying charge‐coupled device camera (iXon 897U; Andor Technology). Actin patch dynamics were monitored by live imaging using cells grown to log phase in synthetic medium and immobilized on 2% agarose in synthetic medium. Images were acquired with laser excitation at 488 and 561 nm for 100 ms each, every 0.5 s. Kymographs representing single endocytic events were selected, and line profiles along each kymograph were exported as text files. Images were processed using Elements AR and ImageJ. Using NIS Elements, kymographs representing single patches that were well isolated in time and space were selected and cropped, and a line profile was drawn along the length of the kymograph. To avoid pseudoreplication, only one patch per cell was selected. A text file representing the raw intensity in both channels was exported from NIS Elements. These text files were collected and parsed using code available at https://github.com/CAYdenberg/Actin-disassembly–endocytic‐patch‐lifetime‐analysis. Wild‐type and mutant cells carrying an integrated Arc15‐GFP were transformed with a low‐copy plasmid expressing Cof1‐mRFP (in frame insertion of mRFP as described in Lin et al. [2010]). Analysis of strains containing Cof1‐mRFP without Arc15‐GFP revealed a bleed‐through for mRFP together with our filter sets. The amount of bleed‐through could be described by the equation *y* = 0.2564*x* + 154.24, where *x* is the intensity of the 561 nm (mRFP) channel and *y* is the intensity of the 488 nm (GFP) channel. After subtracting *y* from the raw intensities in the 488 nm channel, a background value was calculated by averaging the first five and last five data points in each channel, and assuming that the background changed linearly between them (due to photobleaching). After subtracting background, all values were normalized to the maximum intensity in the 488 nm channel. These corrected intensity profiles were used to determine the longest streak of positive values in the 488 and 561 nm channels, which were taken to be the lifetimes of Arc15‐GFP and Cof1‐mRFP, respectively. The difference between the beginning of the GFP and mRFP streaks was taken as the delay between Arc15‐GFP and Cof1‐mRFP association (this was allowed to be negative). However, some patches contained very low mRFP signal, presumably due to plasmid loss. Cells in which the maximum corrected mRFP intensity was not at least three times the greatest negative value in this channel were excluded from analysis of cofilin lifetime and delay.

For alignment and averaging, the corrected profiles were aligned based on the beginning of the Arc15‐GFP streak, determined above, and the average intensity at each time point was determined for all profiles for each strain. Cof1‐mRFP profiles were left out of the averaging if they contained low signal, as above. Profiles were included in the averaging until the GFP streak ended; after that, they were not included. The average profile was stopped after two or fewer individual profiles remained.

### Western Blotting

Protein extracts were prepared as described in Adams et al. [1997], from ∼3.0 × 10^7^ cells. Proteins were resolved on 15% SDS‐PAGE gel and transferred to nitrocellulose. Blotting was performed with chicken αCof1 [Okada et al., 2006] at 1:1000, and rabbit αTub2 [Matsuzaki et al., 1988] at 1:3500. HRP‐conjugated secondary antibodies were subsequently added and bands were visualized by enhanced chemiluminescence. Band intensities were measured using ImageJ.

### Phalloidin Staining and Scoring

Images were acquired as described in Graziano et al. [2011], except that rhodamine–phalloidin (Invitrogen) was used. Images of single cells were cropped out of the field in a “blind” manner, i.e., by a different lab member from the one who performed the acquisition. Scoring was performed by a third lab member, using CellBlind (https://github.com/CAYdenberg/cellblind), which presents the images in a randomized order, and allows assignment of scores while blinded from the source strain and filename. Sample images were prepared for presentation purposes using ImageJ and Adobe Photoshop, and were resampled when necessary to obtain publication‐quality pixel density.

## Supporting information

Supporting Information Movie S1. Time lapse imaging of the cell shown in Fig 2A. Arc15‐GFP and Cof1‐mRFP, in an otherwise wild type cell, were imaged at 0.5 s intervals.

Supporting Information Movie S2. Time lapse imaging of the cell shown in Fig 4C. Cof1‐mRFP, in an *aip1Δ* cell, imaged at 0.5 s intervals.

Supporting Information Tables.
