# Supplementary material for: Combinatorial genetic analysis of a network of actin disassembly‐promoting factors
Source: Cytoskeleton (Hoboken). 2015 Aug 22;72(7):349–61. doi: 10.1002/cm.21231 (PMC5014199; doi:10.1002/cm.21231)
Supplement: Supplementary file 3 — Supporting Information Tables. [file CM-72-349-s003.pdf]

TABLE S1

Strain	Genotype	Origin
Strains without fluorescent markers		
DDY902	*Mata ura3-52 leu2-3,112 his3Δ200 ade2-1*	Kozminski et al, 2000
DDY904	*Matα ura3-52 leu2-3,112 his3Δ200 lys2-801*	Kozminski et al, 2000
DDY1435	*Matα ura3-52 leu2-3,112 his3Δ200 ade2-1 twf1::URA3*	Goode et al, 1998
DDY1437	*Mata ura3-52 leu2-3,112 his3Δ200 ade2-1 twf1::URA3*	Goode et al, 1998
PLY29	*Matα ura3-52 his3Δ200 leu2-3,112 lys2-801 cof1-22::LEU2*	Pekka Lapalainen, University of Helsinki
DAY50	*Mata leu2Δ1 trp1Δ63 ura3-52 his3Δ200 aip1Δ::LEU2*	David Amberg, SUNY Upstate Medical University
AJY12	*Mata ura3-52 leu2-3,112 his3Δ200 ade2-1 twf1Δ::HIS3MX6*	DDY902 tranformation
AJY13	*Matα ura3-52 leu2-3,112 his3Δ200 lys2-801 twf1Δ::HIS3MX6*	DDY904 tranformation
AJY19	*Matα ura3-52 leu2-3,112 his3Δ200 lys2-801 cap2Δ::HIS3MX6*	DDY904 tranformation
AJY20	*Mata ura3-52 leu2-3,112 his3Δ200 ade2-1 srv2Δ::HIS3MX6 twf1::URA3*	DDY1435 x CY92
AJY22	*Matα ura3-52 leu2-3,112 his3Δ200 lys2-801 aip1Δ::URA3*	AJY12 x CY70
AJY24	*Matα ura3-52 leu2-3,112 his3Δ200 lys2-801 crn1Δ::LEU2*	ABY945 x CY70
AJY27	*Mata ura3-52 leu2-3,112 his3Δ200 lys2-801 gmf1Δ::kanMX6*	ABY945 x CY70
AJY29	*Mata ura3-52 leu2-3,112 his3Δ200 lys2-801 aip1Δ::URA3 crn1Δ::LEU2*	ABY945 x CY70
AJY31	*Mata ura3-52 leu2-3,112 his3Δ200 lys2-801 aip1Δ::URA3 gmf1Δ::kanMX6*	ABY945 x CY70
AJY33	*Mata ura3-52 leu2-3,112 his3Δ200 lys2-801 crn1Δ::LEU2 gmf1Δ::kanMX6*	ABY945 x CY70
AJY34	*Mata ura3-52 leu2-3,112 his3Δ200 lys2-801 aip1Δ::URA3 crn1Δ::LEU2 gmf1Δ::kanMX6*	ABY945 x CY70
AJY35	*Matα ura3-52 leu2-3,112 his3Δ200 ade2-1 lys2-801 aip1Δ::URA3 twf1Δ::HIS3MX6*	AJY12 x CY70
AJY36	*Mata ura3-52 leu2-3,112 his3Δ200 trp1Δ63 leu2-801 aip1Δ::LEU2 twf1::URA3*	AJY13 x AJY74
AJY38	*Mata ura3-52 leu2-3,112 his3Δ200 leu2-801 crn1Δ::LEU2 twf1Δ::HIS3*	AJY12 x CY70
AJY39	*Matα ura3-52 leu2-3,112 his3Δ200 ade2-1 lys2-801 gmf1Δ::kanMX6 twf1Δ::HIS3MX6*	AJY12 x CY70
AJY40	*Mata ura3-52 leu2-3,112 his3Δ200 ade2-1 leu2-801 gmf1Δ::kanMX6 twf1Δ::HIS3*	AJY12 x CY70
AJY41	*Matα ura3-52 leu2-3,112 his3Δ200 ade2-1 leu2-801 aip1Δ::URA3 crn1Δ::LEU2 twf1Δ::HIS3*	AJY12 x CY70
AJY42	*Matα ura3-52 leu2-3,112 his3Δ200 ade2-1 leu2-801 aip1Δ::URA3 gmf1Δ::kanMX6 twf1Δ::HIS3*	AJY12 x CY70
AJY44	*Matα ura3-52 leu2-3,112 his3Δ200 leu2-801 crn1Δ::LEU2 gmf1Δ::kanMX6 twf1Δ::HIS3*	AJY12 x CY70
AJY46	*Matα ura3-52 leu2-3,112 his3Δ200 lys2-801 ade2-1 aip1Δ::URA3 crn1::LEU2 gmf1Δ::kanMX twf1Δ::HIS3*	AJY12 x CY70
AJY47	*Mata ura3-52 leu2-3,112 his3Δ200 ade2-1 aip1Δ::URA3 crn1Δ::LEU2 gmf1Δ::kanMX6 twf1Δ::HIS3*	AJY12 x CY70
AJY69	*Matα ura3-52 leu2-3,112 his3Δ200 ade2-1 leu2-801 cap2Δ::HIS3MX6 twf1::URA3*	AJY19 x DDY1437
AJY73	*Mata ura3-52 leu2-3,112 his3Δ200 lys2-801 aip1Δ::LEU2 cap2Δ::HIS3MX6 twf1Δ::URA3*	AJY74 x AJY19
AJY74	*Mata ura3-52 leu2-3,112 his3Δ200 trp1Δ63 leu2-801 aip1Δ::LEU2 twf1::URA3*	AJY69 x DAY50
AJY76	*Mata ura3-52 leu2-3,112 his3Δ200 trp1Δ63 leu2-801 aip1Δ::LEU2 cap2Δ::HIS3MX6*	AJY69 x DAY50
AJY78	*Mata ura3-52 leu2-3,112 his3Δ200 crn1Δ::LEU2 srv2Δ::HIS3*	CY70 x CY92
AJY80	*Mata ura3-52 leu2-3,112 his3Δ200 ade2-1 lys2-801 gmf1Δ::kanMX6 srv2Δ::HIS3*	CY70 x CY92
AJY82	*Mata ura3-52 leu2-3,112 his3Δ200 crn1Δ::LEU2 gmf1Δ::kanMX6 srv2Δ::HIS3*	CY70 x CY92
AJY83	*Mata ura3-52 leu2-3,112 his3Δ200 ade2-1 lys2-801 gmf1Δ::kanMX6 srv2Δ::HIS3 twf1::URA3*	DDY1435 x AJY82
AJY84	*Mata ura3-52 leu2-3,112 his3Δ200 lys2-801 crn1Δ::LEU2 cap2Δ::HIS3MX6*	AJY19 x AJY34
AJY86	*Matα ura3-52 leu2-3,112 his3Δ200 lys2-801 gmf1Δ::kanMX6 cap2Δ::HIS3MX6*	AJY19 x AJY34
AJY87	*Mata ura3-52 leu2-3,112 his3Δ200 ade2-1 lys2-801 trp1Δ63 cap2Δ::HIS3MX6 crn1Δ::LEU2 twf1::URA3*	AJY33 x AJY69
AJY88	*Matα ura3-52 leu2-3,112 his3Δ200 lys2-801 cap2Δ::HIS3MX6 gmf1Δ::kanMX6 twf1::URA3*	AJY33 x AJY69
AJY89	*Mata ura3-52 leu2-3,112 his3Δ200 ade2-1 lys2-801 trp1Δ63 cap2Δ::HIS3MX6 crn1Δ::LEU2 gmf1Δ::kanMX6*	AJY33 x AJY69
AJY91	*Mata ura3-52 leu2-3,112 his3Δ200 ade2-1 lys2-801 trp1Δ63 cap2Δ::HIS3MX6 crn1Δ::LEU2 gmf1Δ::kanMX6 twf1::URA3*	AJY33 x AJY69
CY5	*Mata ura3-52 leu2-3,112 his3Δ200 ade2-1 aip1Δ::URA3*	DDY902 tranformation
CY24	*Mata ura3-52 leu2-3,112 his3Δ200 ade2-1 crn1Δ::LEU2*	DDY902 tranformation
CY38	*Matα ura3-52 leu2-3,112 his3Δ200 lys2-801 gmf1Δ::kanMX*	CY18 x CY24
CY41	*Matα ura3-52 leu2-3,112 his3Δ200 lys2-801 gmf1Δ::kanMX crn1Δ::LEU2*	CY18 x CY24
CY45	*Matα ura3-52 leu2-3,112 his3Δ200 lys2-801 crn1::LEU2*	CY18 x CY24
CY54	*Matα ura3-52 leu2-3,112 his3Δ200 lys2-801 aip1Δ::URA3*	CY5 x CY18
CY56	*Mata ura3-52 leu2-3,112 his3Δ200 aim7Δ::kanMX aip1Δ::URA3*	CY5 x CY18
CY68	*Matα ura3-52 leu2-3,112 his3Δ200 lys2-801 aip1Δ::URA3 crn1Δ::LEU2*	CY5 x CY41
CY69	*Mata ura3-52 leu2-3,112 his3Δ200 ade2-1 aip1Δ::URA3 crn1Δ::LEU2*	CY5 x CY41
CY70	*Matα ura3-52 leu2-3,112 his3Δ200 lys2-801 aip1Δ::URA3 gmf1Δ::kanMX6 crn1Δ::LEU2*	CY5 x CY41
CY92	*Mata ura3-52 leu2-3,112 his3Δ200 ade2-1 srv2Δ::HIS3MX6*	DDY902 tranformation
CY315	*Mata ura3-52 leu2-3,112 his3Δ200 lys2-801 crn1Δ::NatMX twf1Δ::HIS3*	AJY38 transformation
CY317	*Matα ura3-52 leu2-3,112 his3Δ200 ade2-1 lys2-801 aip1Δ::URA3 crn1::NatMX twf1Δ::HIS3*	AJY41 transformation
CY319	*Matα ura3-52 leu2-3,112 his3Δ200 lys2-801 crn1Δ::NatMX gmf1Δ::kanMX twf1Δ::HIS3*	AJY44 transformation
CY321	*Matα ura3-52 leu2-3,112 his3Δ200 lys2-801 ade2-1 aip1Δ::URA3 crn1Δ::NatMX gmf1Δ::kanMX twf1Δ::HIS3*	AJY46 transformation
CY326	*Matα ura3-52 leu2-3,112 his3Δ200 lys2-801 gmf1Δ::kanMX crn1Δ::NatMX*	CY41 transformation
CY328	*Matα ura3-52 leu2-3,112 his3Δ200 lys2-801 crn1Δ::NatMX*	CY45 transformation
CY332	*Matα ura3-52 leu2-3,112 his3Δ200 lys2-801 aip1Δ::URA3 crn1Δ::NatMX*	CY68 transformation
CY334	*Matα ura3-52 leu2-3,112 his3Δ200 lys2-801 aip1Δ::URA3 gmf1Δ::kanMX crn1Δ::NatMX*	CY70 transformation
Patch dynamics strains		
DDY2752	*Matα ura3-52 leu2-3,112 his3Δ200 lys2-801 ARC15-GFP::HIS3*	Kaksonen et al, 2003
CY18	*Matα ura3-52 leu2-3,112 his3Δ200 lys2-801 ARC15-GFP::HIS3 gmf1Δ::kanMX6*	DDY2752 transformation
CY42	*Mata ura3-52 leu2-3,112 his3Δ200 ade2-1 ARC15-GFP::HIS3*	CY18 x CY24
CY47	*Matα ura3-52 leu2-3,112 his3Δ200 lys2-801 ARC15-GFP::HIS3 crn1Δ::LEU2 gmf1Δ::kanMX*	CY18 x CY24
CY53	*Matα ura3-52 leu2-3,112 his3Δ200 ARC15-GFP::HIS3 crn1Δ::LEU2*	CY18 x CY24
CY59	*Mata ura3-52 leu2-3,112 his3Δ200 ade2-1 ARC15-GFP::HIS3 aip1Δ::URA3 gmf1Δ::kanMX*	CY5 x CY18
CY258	*Matα ura3-52 leu2-3,112 his3Δ200 ade2-1 ARC15-GFP::HIS3 aip1Δ::URA3 crn1Δ::LEU2 gmf1Δ::kanMX*	CY41 x CY59
CY259	*Matα ura3-52 leu2-3,112 his3Δ200 lys2-801 ARC15-GFP::HIS3 [CEN/ARS LEU2 COF1::mRFP]*	DDY2752 transformation
CY260	*Matα ura3-52 leu2-3,112 his3Δ200 lys2-801 ARC15-GFP::HIS3 gmf1Δ::kanMX [CEN/ARS LEU2 COF1::mRFP]*	CY18
CY261	*Matα ura3-52 leu2-3,112 his3Δ200 ade2-1 ARC15-GFP::HIS3 aip1Δ::URA3 gmf1Δ::kanMX [CEN/ARS LEU2 COF1::mRFP]*	CY5 x CY18, transformation
CY262	*Mata ura3-52 leu2-3,112 his3Δ200 lys2-801 ARC15-GFP::HIS3 aip1Δ::URA3 [CEN/ARS LEU2 COF1::mRFP]*	CY5 x CY18, transformation
CY266	*Matα ura3-52 leu2-3,112 his3Δ200 ade2-1 lys2-801 ARC15-GFP::HIS3 aip1Δ::URA3 crn1Δ::LEU2*	DDY2752 x CY69
CY268	*Matα ura3-52 leu2-3,112 his3Δ200 lys2-801 ARC15-GFP::HIS3 aip1Δ::URA3 crn1Δ::LEU2 gmfΔ::kanMX twf1Δ::HIS3*	AJY47 x CY47
CY274	*Mata ura3-52 leu2-3,112 his3Δ200 ade2-1 lys2-801 ARC15-GFP::HIS3 crn1Δ::LEU2 twf1Δ::HIS3*	AJY12 x CY268
CY277	*Mata ura3-52 leu2-3,112 his3Δ200 lys2-801 gmf1Δ::kanMX crn1Δ::LEU2 twfΔ::HIS3 ARC15-GFP::HIS3*	AJY12 x CY268
CY278	*Mat? ura3-52 leu2-3,112 his3Δ200 ARC15-GFP::HIS3 aip1Δ::URA3 crn1Δ::LEU2 twf1Δ::HIS3*	AJY12 x CY268
CY279	*Matα ura3-52 leu2-3,112 his3Δ200 lys2-801 ARC15-GFP::HIS3 gmf1Δ::kanMX twf1Δ::HIS3 [CEN/ARS LEU2 COF1::mRFP]*	AJY12 x CY268, tranformation
CY280	*Mata ura3-52 leu2-3,112 his3Δ200 ARC15-GFP::HIS3 aip1Δ::URA3 twf1Δ::HIS3 [CEN/ARS LEU2 COF1::mRFP]*	AJY12 x CY268, tranformation
CY281	*Mata ura3-52 leu2-3,112 his3Δ200 lys2-801 ARC15-GFP::HIS3 aip1Δ::URA3 gmfΔ::kanMX twfΔ::HIS3 [CEN/ARS LEU2 COF1::mRFP]*	AJY12 x CY268, tranformation
CY282	*Matα ura3-52 leu2-3,112 his3Δ200 ARC15-GFP::HIS3 twf1Δ::HIS3 [CEN/ARS LEU2 COF1::mRFP]*	AJY12 x CY268, tranformation
CY303	*Matα ura3-52 leu2-3,112 his3Δ200 ARC15-GFP::HIS3 crn1Δ::NatMX [CEN/ARS LEU2 COF1::mRFP]*	CY53, successive transformations
CY304	*Matα ura3-52 leu2-3,112 his3Δ200 lys2-801 ARC15-GFP::HIS3 crn1Δ::NatMX gmf1Δ::kanMX [CEN/ARS LEU2 COF1::mRFP]*	CY47, successive transformations
CY305	*Mata ura3-52 leu2-3,112 his3Δ200 ade2-1 lys2-801 ARC15-GFP::HIS3 crn1Δ::NatMX twf1Δ::HIS3 [CEN/ARS LEU2 COF1::mRFP]*	CY274, successive transformations
CY306	*Matα ura3-52 leu2-3,112 his3Δ200 ade2-1 ARC15-GFP::HIS3 aip1Δ::URA3 crn1Δ::NatMX gmf1Δ::kanMX [CEN/ARS LEU2 COF1::mRFP]*	CY258, successive transformations
CY307	*Matα ura3-52 leu2-3,112 his3Δ200 ade2-1 lys2-801 ARC15-GFP::HIS3 aip1Δ::URA3 crn1Δ::NatMX [CEN/ARS LEU2 COF1::mRFP]*	CY266, successive transformations
CY308	*Matα ura3-52 leu2-3,112 his3Δ200 lys2-801 ARC15-GFP::HIS3 aip1Δ::URA3 crn1Δ::NatMX gmf1Δ::kanMX twf1Δ::HIS3 [CEN/ARS LEU2 COF1::mRFP]*	CY268, successive transformations
CY309	*Mata ura3-52 leu2-3,112 his3Δ200 lys2-801 ARC15-GFP::HIS3 crn1Δ::NatMX gmf1Δ::kanMX twf1Δ::HIS3 [CEN/ARS LEU2 COF1::mRFP]*	CY277, successive transformations
CY310	*Mat? ura3-52 leu2-3,112 his3Δ200 ARC15-GFP::HIS3 aip1Δ::URA3 crn1Δ::NatMX twf1Δ::HIS3 [CEN/ARS LEU2 COF1::mRFP]*	CY278, successive transformations
CY384	*ura3-52 leu2-3,112 his3Δ200 lys2-801 cof1-22::LEU2 ARC15-GFP::HIS3*	CY42 x PLY29

**Plasmids**		
pBJ1807	*CEN/ARS LEU2 COF1::mRFP*	Lin et al, 2010
pDD424	*CEN/ARS LEU2 GAL1/10*	David Drubin, UC Berkeley
pDD416	*CEN/ARS LEU2 GAL1/10-COF1*	David Drubin, UC Berkeley

TABLE S2

**Raw growth rates (1/min)**							
		Experiment 1	Experiment 2	Experiment 3	Experiment 4	Average	
AJY91	cap2 crn1 gmf1 twf1						
	25C	0.004126921	0.004041301	0.002230429	0.002797209	0.003298965	
	30C	0.004901796	0.004786605	0.001722267	0.001963259	0.003343482	
	34C	0.003157425	0.003595734	0.002739883	0.003418863	0.003227976	
	37C	0.003295008	0.003838567	0.002798129	0.002489394	0.003105275	
AJY12	twf1						
	25C	0.005935799	0.006450542	0.004430749	0.004435013	0.005313026	
	30C	0.012309965	0.005980747	0.005362484	0.005148912	0.007200527	
	34C	0.007122367	0.00708849	0.005653865	0.006364206	0.006557232	
	37C	0.006586434	0.007137438	0.00603367	0.005484523	0.006310516	
AJY78	crn1 srv2						
	25C	0.002850359	0.002891926	0.004561359	0.005084374	0.003847005	
	30C	0.00511364	0.003968916	0.001838169	0.00189931	0.003205009	
	34C	0.002714028	0.002982048	0.001205901	0.00119574	0.002024429	
	37C	0.013022411	0.013366878	3.48E-05	2.75E-05	0.006612896	
AJY16	srv2						
	25C	0.002886762	0.002876379	0.002400637	0.002516282	0.002670015	
	30C	0.004154782	0.003826479	0.00220676	0.001864529	0.003013138	
	34C	0.001204555	0.004736268	2.26E-05	0.001307127	0.001817638	
	37C	0.001411054	0.001630754	0.000409954	0.000441237	0.00097325	
AJY19	cap2						
	25C	0.004900796	0.005215034	0.003420694	0.003686099	0.004305656	
	30C	0.00533942	0.0061679	0.004055436	0.003683028	0.004811446	
	34C	0.005246976	0.005659248	5.14288E-05	0.003545301	0.003625738	
	37C	0.003217501	0.005450208	0.002651885	0.001826227	0.003286455	
AJY76	aip1 cap2						
	25C	0.003659472	0.003673151	0.002939784	0.002750108	0.003255629	
	30C	0.00337665	0.003303516	0.000773258	0.000697008	0.002037608	
	34C	0.005087398	0.003033931	0.000712525	1.71E-05	0.002212739	
	37C	0.000594292	0.000535932	2.64E-05	0	0.000289157	
AJY38	crn1 twf1						
	25C	0.005110681	0.005002968	0.003922878	0.004167619	0.004551037	
	30C	0.00551098	0.008123051	0.005778068	0.005420412	0.006208128	
	34C	0.00682147	0.006634108	0.006323178	0.005961459	0.006435054	
	37C	0.006007287	0.006134024	0.005000664	0.005986193	0.005782042	
AJY73	aip1 cap2 twf1						
	25C	0.005005806	0.004066184	0.003006429	0.002978514	0.003764233	
	30C	0.003561456	0.003970046	0.001903462	0.001816634	0.0028129	
	34C	0.002522949	0.005547742	0.002214837	3.65E-05	0.002580507	
	37C	0	0.000938	0.002082872	0	0.000755218	
AJY33	crn1 gmf1						
	25C	0.00413529	0.007528425	0.004162193	0.004271932	0.00502446	
	30C	0.005104195	0.007554949	0.005676723	0.00560401	0.005984969	
	34C	0.003643641	0.000564288	0.001898583	0.002586354	0.002173217	
	37C	0.000745776	0.000409709	0.001196209	0.002175501	0.001131799	
AJY31	aip1 gmf1						
	25C	0.005296997	0.005698017	0.004930974	0.004028181	0.004988542	
	30C	0.006055924	0.007663344	0.005627871	0.005601731	0.006237218	
	34C	0.00706285	0.00710077	0.006268024	0.006059556	0.0066228	
	37C	0.007333446	0.015911957	0.004000032	0.004362895	0.007902083	
AJY36	aip1 twf1						
	25C	0.005804258	0.006464988	0.00447739	0.004828598	0.005393809	
	30C	0.005026655	0.009859124	0.006066611	0.00551415	0.006616635	
	34C	0.006887659	0.00683093	0.006941243	0.007318479	0.006994578	
	37C	0.005853796	0.005055958	0.005023744	0.004968571	0.005225517	
AJY34	aip1 crn1 gmf1						
	25C	0.005572078	0.006773734	0.004361072	0.004185003	0.005222972	
	30C	0.00503954	0.007875877	0.004766477	0.003924622	0.005401629	
	34C	0.01144275	0.005896652	0.003793208	0.004738209	0.006467705	
	37C	0.006626971	0.006884265	0.004209588	0.004041293	0.005440529	
AJY89	cap2 crn1 gmf1						
	25C	0.004097003	0.004168245	0.001845314	0.002740093	0.003212664	
	30C	0.00429183	0.004420321	0.00266112	0.002543923	0.003479299	
	34C	0.00303475	0.003593252	0.00232155	0.003127602	0.003019289	
	37C	0.001099668	0.002100846	0.001312	0.001009117	0.001380408	
AJY88	cap2 gmf1 twf1						
	25C	0.004564461	0.00470708	0.003229673	0.003461327	0.003990635	
	30C	0.011101664	0.005250412	0.003403773	0.003322027	0.005769469	
	34C	0.005555769	0.005851806	0.004312088	0.004281624	0.005000322	
	37C	0.003204347	0.004145903	0.002523348	0.001922771	0.002949092	
AJY87	cap2 crn1 twf1						
	25C	0.004826305	0.005278456	0.004036993	0.003864698	0.004501613	
	30C	0.007150967	0.005591934	0.00280236	0.002397529	0.004485698	
	34C	0.005192924	0.005738401	0.003924308	0.003177218	0.004508213	
	37C	0.003902476	0.003587206	0.003129401	0.003539694		
AJY86	cap2 gmf1						
	25C	0.006190875	0.005060146	0.00359171	0.003680412	0.004630786	

	30C	0.006165775	0.007349644	0.003723759	0.003719485	0.005239666	
	34C	0.006207697	0.006190862	0.004119428	0.00467783	0.005298954	
	37C	0.005127579	0.003910769	0.002592592	0.002631465	0.003565601	
AJY84	cap2 crn1						
	25C	0.004399965	0.004370645	0.003395534	0.00295814	0.003781071	
	30C	0.005768457	0.007156208	0.002576938	0.002754196	0.00456395	
	34C	0.005812669	0.005696809	0.003301658	0.003432113	0.004560812	
	37C	0.006736287	0.005560174	0.002330612	0.001937648	0.00414118	
AJY83	gmf1 srv2 twf1						
	25C	0.004269753	0.003774825	0.002282958	0.002217071	0.003136152	
	30C	0.008476674	0.008133672	0.001748682	0.001936492	0.00507388	
	34C	1.29E-05	0.002294348	0.000909856	0.00105377	0.001067721	
	37C	0.001960471	0.001457417	1.09E-05	0.000396545	0.000956336	
AJY82	crn1 gmf1 srv2						
	25C	0.002613724	0.001992085	0.002017293	0.002054442	0.002169386	
	30C	0.003381104	0.001838893	0.001531764	0.001821371	0.002143283	
	34C	0.00343324	0.00320637	0.001354888	0.00141579	0.002352572	
	37C	0.003531363	0.002800191	0.000476667	0.001001935	0.001952539	
AJY80	gmf1 srv2						
	25C	0.003055359	0.002861196	0.003342569	0.003601498	0.003215156	
	30C	0.003861023	0.003746426	0.002341647	0.002217039	0.003041534	
	34C	0.003030847	0.002956564	0.001647728	1.62472E-05	0.001912847	
	37C	0.001482742	0.007949056	0.002821	0.00088011	0.003283227	
AJY29	aip1 crn1						
	25C	0.005467955	0.010434575	0.005236013	0.004774197	0.006478185	
	30C	0.007273791	0.008014089	0.006005096	0.00568616	0.006744784	
	34C	0.007555656	0.008245225	0.00606068	0.00598953	0.006962773	
	37C	0.006842607	0.011836118	0.00499354	0.005121534	0.00719845	
AJY42	aip1 gmf1 twf1						
	25C	0.004868396	0.00445578	0.003386223	0.003031281	0.00393542	
	30C	0.004830089	0.004434733	0.002928543	0.00345611	0.003912369	
	34C	0.008573705	0.006783413	0.002519337	3.39847E-05	0.00447761	
	37C	0.002690875	0.004100346	0.000903489	0.000805596	0.002125077	
AJY41	aip1 crn1 twf1						
	25C	0.004177897	0.004092516	0.003236853	0.00295042	0.003614422	
	30C	0.005329343	0.005332491	0.003120308	0.002897255	0.004169849	
	34C	0.004565789	0.004827732	0.003442098	0.003633272	0.004117223	
	37C	0.003916562	0.003350992	0.00177269	0.003304697	0.003086235	
AJY40	gmf1 twf1						
	25C	0.004287658	0.003999019	0.003109053	0.003491891	0.003721905	
	30C	0.005164045	0.008390632	0.002684252	0.002754616	0.004748386	
	34C	0.005399846	0.00562001	0.005306014	0.005143784	0.005367414	
	37C	0.005136213	0.007776434	0.002671084	0.003232379	0.004704028	
AJY47	aip1 crn1 gmf1 twf1						
	25C	0.005054667	0.003973681	0.002497807	0.002401023	0.003481795	
	30C	0.003950696	0.004995762	0.001469795	0.001675321	0.003022894	
	34C	0.003387673	0.004071465	0.001256985	1.65925E-05	0.002183179	
	37C	0.000962967	0.002169439	0.000203	0.000747754	0.00102079	
AJY44	crn1 gmf1 twf1						
	25C	0.004597788	0.004178339	0.003946678	0.003867505	0.004147578	
	30C	0.006297716	0.009142114	0.004679576	0.004568393	0.00617195	
	34C	0.006552121	0.007063002	0.005863291	0.004446889	0.005981326	
	37C	0.005396308	0.006248925	0.004174604	0.004945294	0.005191283	
AJY20	srv2 twf1						
	25C	0.002583062	0.002498102	0.002090458	0.001985063	0.002289171	
	30C	0.003299347	0.002897405	0.001919145	0.001361587	0.002369371	
	34C	0.011351258	0.00913665	1.99E-05	0.001659729	0.005541884	
	37C	0.001302062	0.001377102	6.12E-05	0.000106305	0.000711671	
AJY22	aip1						
	25C	0.005624523	0.00605911	0.005161216	0.004830865	0.005418929	
	30C	0.007313001	0.005873139	0.006357052	0.006371487	0.00647867	
	34C	0.007724461	0.007952002	0.006867259	0.006631471	0.007293798	
	37C	0.005723894	0.005972069	0.006646945	0.00531307	0.005913995	
AJY69	cap2 twf1						
	25C	0.004607068	0.004503142	0.003043623	0.003117705	0.003817885	
	30C	0.005254231	0.004162811	0.002498687	0.002143511	0.00351481	
	34C	0.003498758	0.006351608	0.002644596	5.12296E-05	0.003136548	
	37C	0.004384301	0.00313615	0.002445368	0.002344134	0.003077488	
AJY24	crn1						
	25C	0.005199099	0.004952297	0.004335204	0.004056724	0.004635831	
	30C	0.005695796	0.005408769	0.005623961	0.005498831	0.005556839	
	34C	0.005443587	0.005249466	0.004400976	0.004097723	0.004797938	
	37C	0.003613908	0.001650203	0.002699199	0.002276545	0.002559964	
AJY27	gmf1						
	25C	0.004926625	0.005127622	0.003279926	0.003836144	0.004292579	
	30C	0.005611794	0.00551823	0.005827188	0.005082034	0.005509812	
	34C	0.007124746	0.00660973	0.00640753	0.005788044	0.006482513	
	37C	0.007012098	0.007560188	0.004066557	0.004382224	0.005755267	
AJY2	WT						
	25C	0.005575211	0.005807452	0.004229659	0.004125407	0.004934432	

	30C	0.009429935	0.007622639	0.005063216	0.004967869	0.006770915	
	34C	0.007058077	0.007354086	0.004942611	0.005950914	0.006326422	
	37C	0.00598575	0.006113966	0.006140575	0.005163708	0.005851	

**SUMMARY 7 Lowest temperature necessary to see a ratio of <0.5 compared to the base strain ~ Ratio at that temperature**							
	aip1	cap2	crn1	gmf1	srv2	twf1	
WT	37C ~ 1.01076649337	37C ~ 0.561691230631	37C ~ 0.437525868976	37C ~ 0.983638180808	30C ~ 0.445011879673	37C ~ 1.07853640739	
aip1		30C ~ 0.314510243403	37C ~ 1.21718911812	37C ~ 1.33616669748	ND	37C ~ 0.88358507097	
cap2	30C ~ 0.423491815142		37C ~ 1.2600750459	37C ~ 1.08493832375	ND	37C ~ 0.93641568678	
crn1	37C ~ 2.81193425102	37C ~ 1.61767144164		34C ~ 0.452948016419	34C ~ 0.421937351004	37C ~ 2.25864213898	
gmf1	37C ~ 1.37301759297	37C ~ 0.619537096174	34C ~ 0.335242932428		34C ~ 0.295077957813	37C ~ 0.817343088398	
srv2	ND	ND	37C ~ 6.7946549434	37C ~ 3.37346811546		37C ~ 0.73123180869	
twf1	37C ~ 0.828064938427	30C ~ 0.488132326981	37C ~ 0.916254989439	37C ~ 0.745426731133	25C ~ 0.430860183578		
aip1 cap2			ND	ND	ND	37C ~ 2.61179130653	
aip1 crn1		ND		37C ~ 0.755791793921	ND	37C ~ 0.428736096963	
aip1 gmf1		ND	37C ~ 0.688493096598		ND	37C ~ 0.268926134345	
aip1 twf1		30C ~ 0.425125384731	37C ~ 0.590608566071	37C ~ 0.406672947066	ND		
cap2 crn1	ND			37C ~ 0.333336794504	ND	37C ~ 0.854754953816	
cap2 gmf1	ND		37C ~ 0.387145856537		ND	37C ~ 0.827095360144	
cap2 twf1	37C ~ 0.245400774479		37C ~ 1.15018939011	37C ~ 0.958278963372	ND		
crn1 gmf1	37C ~ 4.80697584266	37C ~ 1.21965830939			25C ~ 0.431765005593	37C ~ 4.58675427058	
crn1 srv2	ND	ND		37C ~ 0.295262307704		ND	
crn1 twf1	37C ~ 0.533762163955	37C ~ 0.612187585862		37C ~ 0.897828613144	ND		
gmf1 srv2	ND	ND	37C ~ 0.5947011888			37C ~ 0.291279418085	
gmf1 twf1	37C ~ 0.451756819024	37C ~ 0.626929211192	37C ~ 1.10358256834		34C ~ 0.198926466165		
srv2 twf1	ND	ND	ND	34C ~ 0.192663821876			
aip1 cap2 twf1			ND	ND	ND		
aip1 crn1 gmf1		ND			ND	34C ~ 0.337550794198	
aip1 crn1 twf1		ND		37C ~ 0.330755732247	ND		
aip1 gmf1 twf1		ND	34C ~ 0.487576834867		ND		
cap2 crn1 gmf1	ND				ND	37C ~ 2.24953424088	
cap2 crn1 twf1	ND			37C ~ 0.877271935816	ND		
cap2 gmf1 twf1	ND		37C ~ 1.05295943184		ND		
crn1 gmf1 srv2	ND	ND				ND	
crn1 gmf1 twf1	30C ~ 0.48977934404	37C ~ 0.598170943395			ND		
gmf1 srv2 twf1	ND	ND	ND				

TABLE S3

Relevant Genotype	Strain	Arc15S GFP lifetime (s)*	Cof1S mRFP lifetime (s)*
ARC15-GFP	DDY2752	18.7 ± 5.2	
ARC15-GFP cof1-22	CY384	47.1 ± 14.3	
ARC15-GFP [COF1::mRFP]	CY259	24.8 ± 6.6	21.4 ± 8.9
ARC15-GFP [vector]	DDY2752 pRS415	21.6 ± 6.2	
[COF1::mRFP]	DDY904 pBJ1807		21.6 ± 9.2
ARC15-GFP [COF1::mRFP] aip1Δ	CY262	31.0 ± 9.0	26.9 ± 11.1
ARC15-GFP [COF1::mRFP] crn1Δ	CY303	30.6 ± 10.5	24.8 ± 10.9
ARC15-GFP [COF1::mRFP] gmf1Δ	CY260	27.8 ± 8.8	21.2 ± 9.9
ARC15-GFP [COF1::mRFP] twf1Δ	CY282	29.4 ± 8.6	23.4 ± 10.8
ARC15-GFP [COF1::mRFP] aip1Δ crn1Δ	CY307	43.0 ± 16.0	40.1 ± 16.6
ARC15-GFP [COF1::mRFP] aip1Δ gmf1Δ	CY261	36.5 ± 11.8	31.6 ± 11.9
ARC15-GFP [COF1::mRFP] aip1Δ twf1Δ	CY280	32.8 ± 9.7	26.5 ± 9.2
ARC15-GFP [COF1::mRFP] crn1Δ gmf1Δ	CY304	37.6 ± 19.8	26.4 ± 16.9
ARC15-GFP [COF1::mRFP] crn1Δ twf1Δ	CY305	40.4 ± 15.4	31.2 ± 14.1
ARC15-GFP [COF1::mRFP] gmf1Δ twf1Δ	CY279	33.0 ± 12.4	24.9 ± 13.2
ARC15-GFP [COF1::mRFP] aip1Δ crn1Δ gmf1Δ	CY306	36.4 ± 8.9	26.9 ± 10.1
ARC15-GFP [COF1::mRFP] aip1Δ crn1Δ twf1Δ	CY310	50.4 ± 15.1	35.0 ± 11.9
ARC15-GFP [COF1::mRFP] aip1Δ gmf1Δ twf1Δ	CY281	37.7 ± 13.3	30.8 ± 11.3
ARC15-GFP [COF1::mRFP] crn1Δ gmf1Δ twf1Δ	CY309	52.3 ± 15.7	38.8 ± 16.1
ARC15-GFP [COF1::mRFP] aip1Δ crn1Δ gmf1Δ twf1Δ	CY308	68.7 ± 30.9	58.2 ± 31.3
* Data are means ± standard deviation			

LEGENDS TO SUPPLEMENTARY TABLES AND MOVIES

Table S1. Details of yeast strain construction.

Table S2. Comparison of growth rates of wild type and mutant strains. *Top:* Raw growth rates for all mutant strains shown in Fig 1. *Bottom*: Summary of minimal temperature required to see at least a 2-fold decrease in the growth rate compared to the base strain.

Table S3. Arc15-GFP and Cof1-mRFP mean lifetimes in wild type and mutant strains. Data from scatter plots in Fig 2E and Fig 3B.

Movie S1. Time lapse imaging of the cell shown in Fig 2A. Arc15-GFP and Cof1-mRFP, in an otherwise wild type cell, were imaged at 0.5 s intervals.

Movie S2. Time lapse imaging of the cell shown in Fig 4C. Cof1-mRFP, in an *aip1*Δ cell, imaged at 0.5 s intervals.
